# Level of tuberculosis-related stigma and associated factors in Ugandan communities

**DOI:** 10.1371/journal.pone.0313750

**Published:** 2025-01-24

**Authors:** Derrick Kimuli, Florence Nakaggwa, Norah Namuwenge, Vincent Kamara, Mabel Nakawooya, Geofrey Amanya, Philip Tumwesigye, Daniel Mwehire, Deus Lukoye, Miriam Murungi, Seyoum Dejene, Jaffer Byawaka, Norbert Mubiru, Stavia Turyahabwe, Barbara Amuron, Daraus Bukenya

**Affiliations:** 1 Social & Scientific Systems, Inc., a DLH Holdings Company / United States Agency for International Development Strategic Information Technical Support Activity, Kampala, Uganda; 2 Institute of Public Health and Management, Clarke International University, Kampala, Uganda; 3 National Tuberculosis and Leprosy Program, Ministry of Health Uganda, Nakasero, Kampala, Uganda; 4 United States Agency for International Development Local Partner Health Services–TB, Infectious Diseases Institute, College of Health Sciences, Makerere University, Kampala, Uganda; 5 The United States Agency for International Development Uganda, Kampala, Uganda; 6 The United States Centers for Disease Control and Prevention, Kampala, Uganda; University of Melbourne, AUSTRALIA

## Abstract

Tuberculosis (TB) stigma remains a significant barrier to TB control efforts globally, especially in countries with a high TB burden. Studies about TB stigma done in Uganda so far have been limited in scope and focused on data collected health facilities. In this study we report TB related stigma at community level for the period 2021/2022. We used the 2021/22 Lot Quality Assurance Sampling (LQAS) data from a sample of 33,349 participants across 77 districts, to measure TB stigma determine factors associated. We included demographic characteristics, knowledge and participant perspectives as our study variables. Univariable and multivariate logistic regression analyses were performed to identify factors associated with TB stigma. TB stigma was assessed as a categorical variable (below or above the median) due to the skewness of the data when fitting the scores. The data set had equal proportions of males and females. The largest age group was 20–29 years old (38.47%). Most participants were married (62.94%) and had primary level education (65.80%). The TB stigma scores were assigned on a scale from 0 to 30, with an average score of 21.67 (±8.22) and a median score of 24 (19–28). Overall, 45.48% of participants had TB stigma scores above the median. Variations in TB stigma levels were observed across different districts. Factors associated with higher TB stigma included older age, higher education levels, urban residence, and TB knowledge. To reduce TB stigma and misinformation that can make an impact on TB response, community interventions should balance increasing awareness with minimizing fear. These interventions should be well-rounded and context-specific to address disparities within communities and bolster TB control efforts in the country.

## Introduction

Low- and middle-income countries are still disproportionately affected by tuberculosis (TB) where it remains a major public health challenge. This is exacerbated by several other epidemics and emerging diseases [1]. In 2022, about 11 million new TB cases were notified globally, with a significant burden in sub-Saharan Africa [1]. The consequences of TB are far reaching with diverse social, emotional and economic implications on families, relationships and communities [1]. TB stigma, defined as negative attitudes and beliefs about individuals with TB, exacerbates the disease’s impact by hindering early diagnosis, treatment adherence, and social support [2, 3]. Furthermore, stigma not only affects patients’ physical health but also their psychological well-being and quality of life [4]. As with other mental health conditions in communities, the psychological well-being of patients post-TB care—where stigma plays a significant role [2–4]—receives minimal attention, unlike more commonly addressed conditions such as post-TB infections [5, 6].

Despite considerable efforts to combat TB in all its forms, [7], Uganda, a country with a high TB burden, continues to struggle with controlling the spread of the disease [8–10]. Among other challenges, TB stigma significantly undermines public health initiatives related to TB care and support [3, 11]. Therefore, understanding the levels of TB stigma and the associated factors is essential for effective TB control efforts. Previous studies have identified various determinants of TB stigma, including sociodemographic factors such as age, sex, education level, and residence, as well as TB knowledge and awareness [3, 11–14]. However, within the context of Uganda, such studies have primarily been patient-focused and facility-based [3, 11] or limited in scope by a small sample in a single district [14]. This study used pooled Lot Quality Assurance Sampling (LQAS) survey data from 77 districts to assess the levels of TB stigma across different regions in Uganda to identify the factors associated with TB stigma.

## Materials and methods

### Study design, population and setting

The LQAS methodology assesses process quality or conditions within a population using statistical sampling to quickly determine if a specific parameter meets a pre-defined threshold [15, 16]. Ideal for resource-constrained settings, the LQAS approach divides populations into smaller units or "lots" which are classified as "acceptable" or "unacceptable" based on a determined criterion [15, 17, 18]. It estimates overall coverage reliably by collating lot results [15, 19]. The 2021/22 LQAS surveys, cross-sectional studies, included 77 districts divided into 5–7 lots each. Using probability proportionate to size sampling, 19–24 villages per lot were selected, with households chosen randomly for interviews. In this report, we present findings on TB related stigma and knowledge among men women and youth using data collected during the 2021/2022 survey [18–23].

### Study variables and measurements

The LQAS questionnaire is a validated questionnaire designed to collect data on a range of health-related topics, including TB stigma. In the questionnaire, TB stigma, as the study’s dependent variable was measured using a set of 10 validated questions, with responses recorded on a Likert scale. These questions assessed the community’s sentiments on various TB-related issues (see Table 2 in the results). The independent variables included demographic characteristics and TB knowledge factors (signs, symptoms and management of TB). Demographic characteristics included sex, age, marital status, highest education level, and residence. Sex was recorded as a binary variable (male or female), while age was categorized into evenly distributed groups: <20, 20–29, 30–39, 40–49, and ≥50 years for a more reliable statistical analysis. Marital status was classified as single, married, or previously married (including divorced, widowed, and separated). Education level was categorized into four groups: no formal education, primary education, secondary education, and tertiary education. Residence was recorded as urban or rural, but participants hailed from diverse districts within the regions across Uganda. TB knowledge factors were derived from multiple questions about TB transmission, symptoms, prevention, and treatment.

### Data collection and processing

#### Statistical analysis

Participant demographic characteristics and knowledge on various TB aspects were described using frequencies and percentages or means and corresponding standard deviations. TB stigma was scored between 0 and 30 with higher scores indicating higher stigma. TB stigma as a linear outcome was not normally distributed, hence it was categorized into a binary outcome. The categorical measure was based on the median score; participants whose score was above the median were considered to have high stigma while those at the median point or below were considered to have low or no stigma. In the bivariate analysis, categorical data were summarized using frequencies and percentages, while continuous data were summarized using means and standard deviations. The data were compared for associations between TB stigma and the independent variables using the Chi-square test for categorical variables and the t-test or analysis of variance (ANOVA) for continuous variables. Multivariable logistic and linear regression analysis were done with variables that were statistically significant following the chi-square and t-tests/ANOVA respectively at a *p*-value of less than 5%. Unadjusted odds ratios (uORs) and adjusted odds ratios (aORs) were calculated alongside corresponding 95% confidence intervals (CI) as part of the analysis. In the multivariable analysis, variables with a *p*-value less than 5% were regarded as statistically significant. The analysis was performed utilizing STATA version 17 [24]. To examine geographic patterns and relationships between TB stigma and knowledge, spatial analysis was conducted using Quantum Geographic Information System (QGIS), an open-source GIS software [25].

#### Ethical consideration

The study was a secondary analysis of the LQAS survey data, which is publicly available upon reasonable request at the participating districts or projects. Due to this data’s availability, ethical review consideration or informed consent was not required for the study. We obtained permission from the United States Agency for International Development Strategic Information Technical Support Activity, Social & Scientific Systems, Inc., a DLH Holdings Company, to use the anonymized survey datasets. For more information on the LQAS study’s procedures, one can refer to the LQAS reports [17, 18, 26]. To ensure adherence to appropriate reporting guidelines, the study results were reported following acceptable standards and guidelines for observational studies in epidemiology [27].

## Results

### Participant characteristics and the level of TB stigma

Overall, the pooled survey data contained interview records for 47,711 participants. We excluded 14,362 (30.10%) records from the analysis which lacked the TB component among the participants assessed. Table 1 shows participant characteristics and their association with TB stigma. Of the 33,349 records included 16,679 were for female (50.0%) participants. Mean age of the participants was 26.7 (±10.2), the age group of the 20–29-year-olds (38.5%) contributed most participants compared to other age groups. 20,990 (62.9%) were married, while 21,944 (65.8%) had only completed primary level and rural residents were 27,477 (82.39%). The mean household size was 5.4 (±2.9) but most of the participants were from households with members more than the mean household size (56.5%).

**Table 1:** Participants’ characteristics and TB stigma.

Variable	Disaggregation	Frequency, N = 33,349 (% = 100)^¥^	TB Stigma		
			≤Median (Low) (N = 18,182; 54.5)^π^	>Median (High) (N = 15,167; 45.5)^π^	P-value
Sex	Male	16,670 (50.0)	8,979 (53.9)	7,691 (46.1)	0.016
	Female	16,679 (50.0)	9,203 (55.4)	7,476 (44.8)	
Marital Status	Never married	11,001 (33.0)	6,396 (58.1)	4,605 (41.9)	<0.001
	Married	20,990 (62.9)	11,082 (52.8)	9,908 (47.2)	
	Previously married	1,358 (4.3)	704 (51.8)	654 (48.2)	
Age group	10–19 years	9,730 (29.2)	5,936 (61.0)	3,794 (39.0)	<0.001
	20–29 years	12,829 (38.5)	6,815 (53.1)	6,014 (46.9)	
	30+ years	10,790 (32.4)	5,431 (50.3)	5,359 (49.7)	
Education level	None	1,720 (5.2)	1,002 (58.3)	718 (41.7)	<0.001
	Primary	21,944 (65.8)	12,460 (56.8)	9,484 (43.2)	
	Secondary	7,428 (22.3)	3,688 (49.7)	3,740 (50.4)	
	Above secondary	2,257 (6.8)	1,032 (45.7)	1,225 (54.3)	
Residence	Urban	5,872 (17.6)	3,026 (51.5)	2,846 (48.5)	<0.001
	Rural	27,477 (82.4)	15,156 (55.2)	1,2321 (44.8)	
Household size	< = meanhhs	18,842 (56.5)	10,364 (55)	8,478 (45.0)	0.043
	>meanhhs	14,507 (43.5)	7,818 (53.9)	6,689 (46.1)	

hhs–Household size

SD–Standard deviation

^**¥**^ *Column percentages shown*

^**π**^ *Row percentages shown*

TB stigma scores ranged from 0 to 30 with an average score was 21.67 (±8.22) negatively skewed distribution (-1.24), and a kurtosis value of 3.91 indicating that the distribution had heavier tails than a normal distribution, due extremities. The median score was 24 (Interquartile Range [IQR] 19–28). High TB stigma was common among participants who were male (46.14%), previously married (48.16%), aged 30 years and above (49.67%), those with education above secondary (54.28%). High TB stigma was also more common among urban residents (48.47%), and those who lived in households with greater than the mean household size (46.11%). All the background characteristics had a statistically significant association with TB stigma (*p*<0.05).

### TB stigma aspects

Table 2 presents the findings on community perceptions and reactions regarding TB-stigma. A significant proportion of respondents perceived TB-related stigma as a persistent issue, with over half of them (52.5%) believing that community members will treat a person with TB differently for the rest of their lives. Similarly, discomfort around TB patients is widespread, with 52.6% of respondents agreeing that some people felt uncomfortable being close to someone with TB. There was also a notable concern about TB patients interacting with children, as 51.5% of respondents believed that some people did not want TB patients to play with their children.

**Table 2:** Frequency and distribution of community perceptions related to TB stigma.

Question	Response	Frequency	Percentage
**a) If a person has TB, some members of the community will behave differently in relation to that person for the rest of their lives**	Don’t know	3,434	10.3
	Not true at all	3,053	9.2
	Somewhat true	9,359	28.1
	Very True	17,503	52.5
**b) Some people feel uncomfortable when they are close to a person with TB**	Don’t know	3,321	10.0
	Not true at all	2,603	7.8
	Somewhat true	9,872	29.6
	Very True	17,553	52.6
**c) Some people do not want people with TB playing with their children**	Don’t know	3,632	10.9
	Not true at all	3,064	9.2
	Somewhat true	9,495	28.5
	Very True	17,158	51.5
**d) Some people with TB feel hurt with the way other people react when they learn that they have TB**	Don’t know	3,955	11.9
	Not true at all	3,001	9.0
	Somewhat true	10,181	30.5
	Very True	16,212	48.6
**e) Some people with TB lose friends when they share the information that they have the disease**	Don’t know	4,179	12.5
	Not true at all	3,364	10.1
	Somewhat true	10,138	30.4
	Very True	15,668	47.0
**f) Some people with TB are worried about the possibility of having AIDS too**	Don’t know	3,921	11.8
	Not true at all	2,458	7.4
	Somewhat true	8,370	25.1
	Very True	18,600	55.8
**g) Some people with TB fear telling other people about their condition because other people may think they have AIDS too**	Don’t know	3,699	11.2
	Not true at all	2,667	8.0
	Somewhat true	10,146	30.4
	Very True	16,837	50.5
**h) Some people with TB fear telling their families that they have the disease**	Don’t know	3,772	11.3
	Not true at all	4,256	12.8
	Somewhat true	10,650	31.9
	Very True	14,671	45.0
**i) Some people with TB will carefully choose those who they will inform about their condition**	Don’t know	4,255	12.8
	Not true at all	3,062	9.2
	Somewhat true	10,633	31.9
	Very True	15,399	46.2
**j) Some people with TB fear going to TB clinics because other people may see them there**	Don’t know	4,360	13.1
	Not true at all	3,994	12.0
	Somewhat true	10,321	31.0
	Very True	14,674	44.0

Psychological effects were also evident, with 48.6% of respondents acknowledging that TB patients felt hurt by others’ reactions, and 47.0% indicating that TB patients lost friends when they disclose their condition. Fear of association with HIV/AIDS was also common, with 55.8% and 50.5% of respondents, affirming that TB patients worry about being perceived as having AIDS and fear disclosing their condition for this reason. Additionally, a substantial number of TB patients were selective about whom they inform about their condition (46.2%) and avoided TB clinics due to fear of being seen (44.0%).

### Knowledge factors and TB stigma

**Fig 1:**
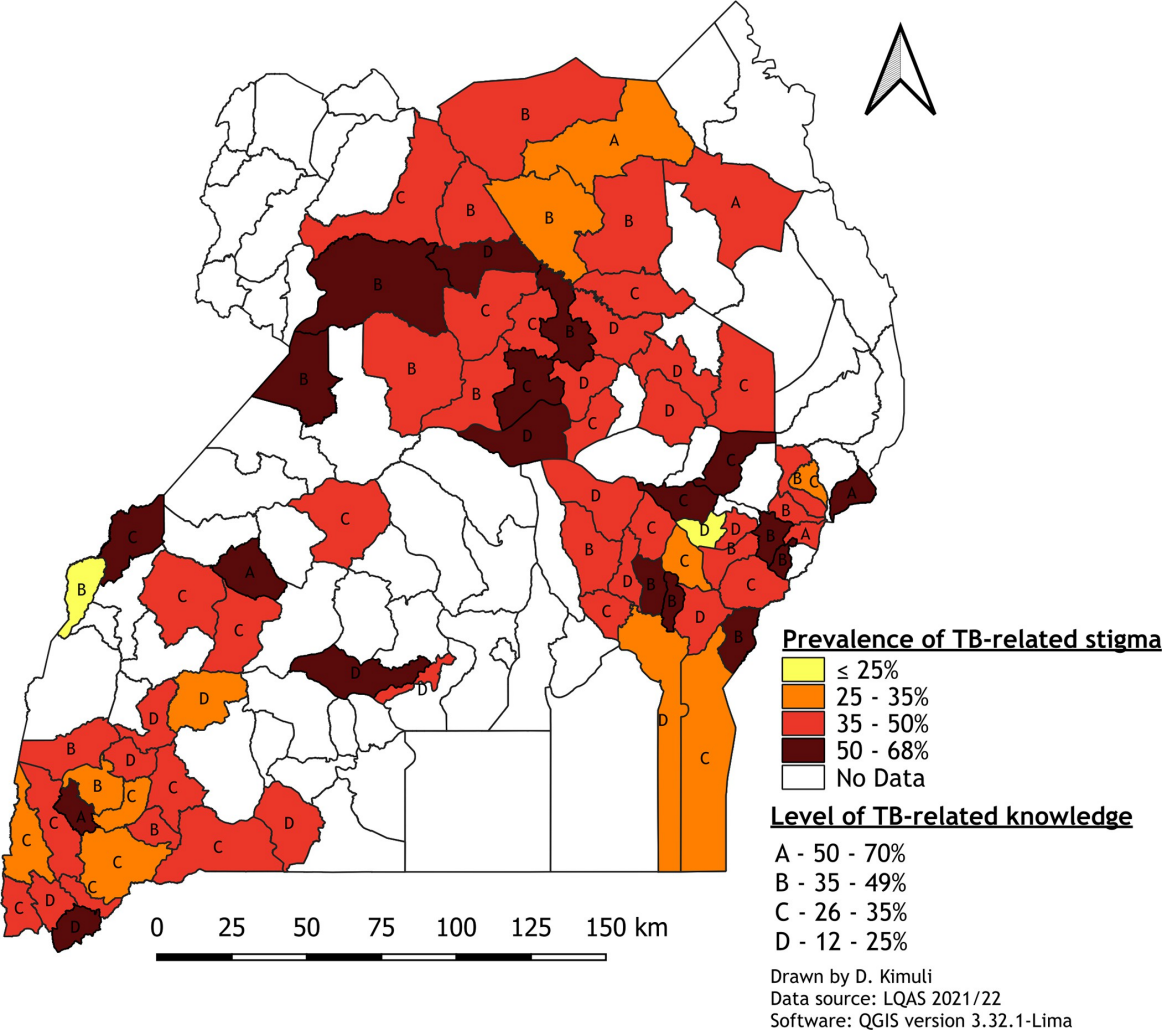
Spatial distribution of TB stigma and knowledge–Uganda. Printed using QGIS under a CC BY license, with permission from Derrick Kimuli, original copyright 2024.

Fig 1 shows the spatial distribution of TB stigma and knowledge in Uganda.

High levels of stigma (indicated by red or dark red) were mostly concentrated in the nothern, east and east-central regions, with prevalence rates ranging from 50% to 68%. Areas with lower stigma (≤35%, shown in yellow or orange) were sparsely distributed, predominantly in the southwestern parts of the country. Regarding TB-related knowledge, the majority of districts were within the mid-range categories (B and C, representing 35–49% and 26–35% knowledge levels, respectively). Districts with high stigma did not consistently correlate with lower knowledge levels; for example, some northern districts with high stigma still exhibited moderate to high knowledge levels (A and B).

Table 3 shows the relationship between TB knowledge and TB stigma. Knowledge of the mode of TB transmission was relatively high among the participants, however, knowledge of some symptoms and consequences of untreated TB was low. For instance, of the 33,349 participants, 79.7% and 87.6% believed that it was possible to have both HIV and TB at the same time and that TB is transmitted through exposure to germs in the air. However, only 15.5%, 12.8%, 16.2% and 19.6%, identified evening fever, sweating at night, loss of appetite and body weakness as possible symptoms to TB. The level of reported high TB stigma was higher among participants who knew that it was possible to have both TB and HIV (49.1%), among those who knew that TB is transmitted through exposure to TB germs in air (47.7%) and only slightly higher among those who did not know that TB could be transmitted through drinking unpasteurized infected milk (44.5%).

**Table 3:** Relationship between TB knowledge and TB stigma.

TB Knowledge aspect	Disaggregation	Frequency, N = 33349 (percentage = 100%)^¥^	TB Stigma		P-value
			≤Median (Low) (N = 18182; 54.52%)^π^	>Median (High) (N = 15167; 45.48%)^π^	
Is it possible for someone to have both TB and HIV at the same time?	No	6,773 (20.3)	4,661 (68.8)	2,112 (31.2)	<0.001
	Yes	26,576 (79.7)	13,521 (50.9)	13,055 (49.1)	
Is TB transmitted through exposure to TB germs in air?	No	4,151 (12.5)	2,911 (70.1)	12,40 (29.9)	<0.001
	Yes	29,198 (87.6)	15,271 (52.3)	13,927 (47.7)	
Is TB transmitted through drinking unpasteurized infected milk	No	29,337 (88.0)	15,955 (54.4)	13,382 (45.6)	0.18
	Yes	4,012 (12.0)	2,227 (55.5)	1,785 (44.5)	
Mentioned “cough for two weeks or more” as a possible TB symptom	No	6,824 (20.5)	4,394 (64.4)	2,430 (35.6)	<0.001
	Yes	26,525 (79.5)	13,788 (52.0)	12,737 (48.0)	
Mentioned pain in chest as a possible TB symptom	No	17,585 (52.7)	10,258 (58.3)	7,327 (41.7)	<0.001
	Yes	15,764 (47.3)	7,924 (50.3)	7,840 (49.7)	
Mentioned “coughing blood” as a possible TB symptom	No	21,207 (63.6)	12,163 (57.4)	9,044 (42.7)	<0.001
	Yes	12,142 (36.4)	6,019 (49.6)	6,123 (50.4)	
Mentioned “unexplained weight loss” as a possible TB symptom	No	24,943 (74.8)	14,282 (57.3)	10,661 (42.7)	<0.001
	Yes	8,406 (25.2)	3,900 (46.4)	4,506 (53.6)	
Mentioned “evening fever” as a possible TB symptom	No	28,180 (84.5)	15,892 (56.4)	12,288 (43.6)	<0.001
	Yes	5,169 (15.5)	2,290 (44.3)	2,879 (55.7)	
Mentioned “sweating at night” as a possible TB symptom	No	29,088 (87.2)	16,326 (56.1)	12,762 (43.9)	<0.001
	Yes	4,261 (12.8)	1,856 (43.6)	2,405 (56.4)	
Mentioned “loss of appetite” as a possible TB symptom	No	27,948 (83.8)	15,785 (56.5)	12,163 (43.5)	<0.001
	Yes	5,401 (16.2)	2,397 (44.4)	3,004 (55.6)	
Mentioned “body weakness” as a possible TB symptom	No	26,826 (80.4)	15,232 (56.8)	11,594 (43.2)	<0.001
	Yes	6,523 (19.6)	2,950 (45.2)	3,573 (54.8)	
Know that TB curable?	No	8,646 (25.9)	5,663 (65.5)	2,983 (34.5)	<0.001
	Yes	24,703 (74.1)	12,519 (50.7)	12,184 (49.3)	
Know that not adhering to TB treatment could lead to “Drug Resistance”	No	19,001 (57.0)	11,079 (58.3)	7,922 (41.7)	<0.001
	Yes	14,348 (43.0)	7,103 (49.5)	7,245 (50.5)	
Know that not adhering to TB treatment could lead to “death”	No	5,276 (15.8)	3,615 (68.5)	1,661 (31.5)	<0.001
	Yes	28,073 (84.2)	14,567 (51.9)	13,506 (48.1)	
Know that not adhering to TB treatment could lead to “remaining infectious”	No	22,914 (68.7)	13,303 (58.2)	9,611 (41.9)	<0.001
	Yes	10,435 (31.3)	4,879 (46.7)	5,556 (53.2)	

^**¥**^Column percentages shown

^**π**^Row percentages shown

High TB stigma was also common among participants who identified coughing blood (50.4%), unexplainable weight loss (53.6%), evening fever (55.7%), sweating at night (56.4%), loss of appetite (55.6%) and body weakness (54.8%) compared to their counterparts who did not identify these symptoms. Furthermore, high TB stigma was more common among participants who mentioned remaining infectious (53.2%) getting resistant TB (50.5%) and death (48.1%) as consequences of not adhering to TB treatment compared to those who did not mention these consequences. All the components of TB knowledge, except for knowledge of TB transmission through drinking unpasteurized infected milk were statistically associated with high TB stigma.

### Multivariate analysis of factors associated with the TB stigma

Table 4 presents the results from the multivariable analysis of factors associated with high TB stigma. In the adjusted analysis, participants aged 20–29 years (aOR: 1.2, 95% CI: 1.1–1.2) and those aged 30 years and older (aOR: 1.2, 95% CI: 1.1–1.3) had increased odds of reporting high TB stigma compared to younger participants. Educational attainment was also associated with higher odds of TB stigma, with participants who had a secondary education (aOR: 1.2, 95% CI: 1.0–1.3) and higher education (aOR: 1.3, 95% CI: 1.1–1.5) reporting 20% and 30% greater odds of high TB stigma, respectively, compared to those with no formal education. Also, participants who knew it was possible to have both TB and HIV simultaneously were 50% more likely to report high TB stigma (aOR: 1.5, 95% CI: 1.4–1.6). Similarly, those who recognized airborne transmission as a mode of TB spread had 40% higher odds of reporting high TB stigma (aOR: 1.4, 95% CI: 1.3–1.5). Participants who mentioned symptoms such as a persistent cough lasting two weeks, unexplained weight loss, evening fever, night sweats, loss of appetite, and body weakness were 10% more likely to report high stigma. Additionally, certain consequences of non-adherence to TB treatment were linked to higher stigma: resistance to TB (aOR: 1.1, 95% CI: 1.0–1.1), the risk of death (aOR: 1.6, 95% CI: 1.5–1.7), and the belief that individuals would remain infectious (aOR: 1.2, 95% CI: 1.2–1.3). Finally, participants who knew that TB was curable had 30% higher odds of reporting high stigma (aOR: 1.3, 95% CI: 1.2–1.4).

**Table 4:** Factors associated with high TB stigma.

Variable	Categorization	Unadjusted OR (95% CI)	P-value	Adjusted OR (95% CI)	P-value
Sex	Male	1	0.016	1	0.624
	Female	0.95 (0.9–1.0)		1.0 (1–1.1)	
Marital Status	Never married	1		1	
	Married	1.2 (1.2–1.3)	<0.001	1.0(1–1.1)	0.728
	*Previously married*	1.3 (1.2–1.4)	<0.001	1.1(1–1.3)	0.133
Age (10-bands)	10–19	1		1	
	20–29	1.38 (1.3–1.5)	<0.001	1.2 (1.1–1.2)	<0.001
	30+	1.54 (1.5–1.6)	<0.001	1.2 (1.1–1.3)	<0.001
Education level	None	1		1	
	Primary	1.1 (1.0–1.2)	0.234	1.0 (0.9–1.2)	0.603
	Secondary	1.4 (1.3–1.6)	<0.001	1.2 (1.0–1.3)	0.015
	Above secondary	1.7 (1.5–1.9)	<0.001	1.3 (1.1–1.5)	<0.001
Residence	Urban	1		1	
	Rural	0.9 (0.8–0.9)	<0.001	0.9 (0.9–1.0)	0.009
Household size	< = meanhhs	1		1	
	>meanhhs	1(1.0–1.1)	0.043	1.0(1.0–1.1)	0.095
Is it possible for someone to have both TB and HIV at the same time?	No	1	<0.001	1	<0.001
	Yes	2.1(2.0–2.3)		1.5 (1.4–1.6)	
Is TB transmitted through exposure to TB germs in air?	No	1	<0.001	1	<0.001
	Yes	2.1(2.0–2.3)		1.4 (1.3–1.5)	
Mentioned “cough for two weeks or more” as a possible TB symptom	No	1	<0.001	1	<0.001
	Yes	1.7 (1.6–1.8)		1.1(1.1–1.2)	
Mentioned pain in chest as a possible TB symptom	No	1	<0.001	1	0.505
	Yes	1.4 (1.3–1.4)		1.0(0.9–1)	
Mentioned “coughing blood” as a possible TB symptom	No	1	<0.001	1	0.456
	Yes	1.4 (1.3–1.4)		1.0(1.0–1.1)	
Mentioned “unexplained weight loss” as a possible TB symptom	No	1	<0.001	1	0.057
	Yes	1.5(1.5–1.6)		1.16(1.0–1.1)	
Mentioned “evening fever” as a possible TB symptom	No	1	<0.001	1	0.001
	Yes	1.6 (1.5–1.7)		1.1 (1.0–1.2)	
Mentioned “sweating at night” as a possible TB symptom	No	1	<0.001	1	0.109
	Yes	1.7(1.6–1.8)		1.1 (1.0–1.2)	
Mentioned “loss of appetite” as a possible TB symptom	No	1	<0.001	1	0.122
	Yes	1.6 (1.5–1.7)		1.1 (1.0–1.1)	
Mentioned “body weakness” as a possible TB symptom	No	1	<0.001	1	0.001
	Yes	1.6 (1.5–1.7)		1.1 (1.0–1.2)	
Know that TB curable?	No	1	<0.001	1	<0.001
	Yes	1.8 (1.8–1.9)		1.3(1.2–1.4)	
Know that not adhering to TB treatment could lead to “Drug Resistance”	No	1	<0.001	1	<0.001
	Yes	1.4 (1.4–1.5)		1.1 (1.0–1.1)	
Know that not adhering to TB treatment could lead to “death”	No	1	<0.001	1	<0.001
	Yes	2 (1.9–2.1)		1.6 (1.5–1.7)	
Know that not adhering to TB treatment could lead to “remaining infectious”	No	1	<0.001	1	<0.001
	Yes	*(1.5–1.7)		1.2 (1.2–1.3)	

uOR: Unadjusted odds ratio; aOR: Adjusted odds ratio.

Findings from a multilevel logistic regression

## Discussion

This study examined reported TB stigma using data from the LQAS survey conducted during 2021/22 across 77 out of 135 districts in Uganda. The findings revealed geographical and demographic disparities in TB stigma. High levels of TB stigma were observed in regions with both low and high levels of TB knowledge, moreover, TB stigma increased with knowledge. The study also found that TB was higher among older participants and those who completed secondary and higher education level but slightly less among rural compared to urban households. Moreover, the study findings also revealed diverse perceptions, misconceptions, and knowledge gaps about TB within the communities.

One in two participants reported high TB stigma, an observation similar to findings from studies in western Uganda [14] and Kampala [3, 11]. Geographical disparities were evident, with southwestern districts and rural households exhibiting the lowest levels of stigma. These patterns are corroborated by similar findings in a study from Kenya [28]. Geography shapes social norms and culture, and Uganda’s diverse geography contributes to its cultural diversity [29], which may partly explain the variations in TB stigma [2]. In districts with high stigma levels, this can compromise TB care seeking and/or frustrate TB treatment adherence and exacerbating disease spread [2, 14]. Without district-based TB prevalence surveys, TB case detection expectations may be skewed. Understanding the geographical and cultural aspects of districts with high stigma can help improve TB planning efforts.

The spatial analysis also showed that some districts with the highest levels of stigma often also had high levels of TB knowledge. Moreover, various aspects of correct TB knowledge were associated with an increase in TB stigma. This finding is intriguing and could demonstrate the paradoxical relationship between TB knowledge and stigma. Several factors could explain the paradox: the type of knowledge disseminated might focus on the severity and contagiousness of TB, inadvertently heightening fear [13]; cultural norms might amplify stigma despite factual knowledge [4, 30]; and incomplete or misleading information might foster stigma. The counterintuitive result suggests that TB knowledge alone may not be sufficient to reduce stigma. Therefore, educational interventions need to provide balanced information that reduces unfounded fears while emphasizing positive treatment outcomes.

Participants who completed secondary or higher education and older participants were more likely to report TB stigma in the community. This finding aligns with research conducted in a western Uganda district, which indicated that higher education levels were associated with increased TB stigma [14]. One possible cause for this correlation is that individuals with higher education levels may have greater awareness of TB and its social implications, making them more perceptive to stigma [31]. Additionally, older individuals might have experienced prolonged exposure to community attitudes towards TB, contributing to heightened stigma perception [4, 30]. Moreover, the present study also found an increase in TB stigma with increase in TB knowledge. Overall, the implications of this finding may speak to a need for interventions that promote understanding and empathy towards TB patients and encourage supportive community environments.

The high agreement with some TB stigma statements reflected deep-seated stigma within communities. A significant proportion of participants felt uncomfortable around TB patients, feared losing friends, and were concerned about being perceived as having AIDS. Such perceptions can deter individuals from seeking timely diagnosis and treatment, exacerbating the spread of TB [4, 13, 30]. Community perceptions and social reactions to TB are critical factors that influence stigma [32]. Therefore, the study findings shed light on the need for a pathway focused on changing community attitudes and reducing discrimination against TB patients and presumptive TB cases. This could foster better treatment outcomes and case detection rates [3, 33].

Finally, the study findings also showed that knowledge gaps and misconceptions about TB transmission and symptoms were prevalent among participants. For instance, many were unaware of the possibility of TB/HIV co-infection or the key symptoms of TB (such as chest pain, coughing blood, night sweats among others) and that not adhering to TB treatment could lead to consequences such as drug resistance. This was associated with higher stigma levels, implying a need to improve TB knowledge in communities [12, 34]. However, the paradoxical relationship between knowledge and stigma underscores the need for educational programs to provide well-rounded information that not only informs but also reduces fear and stigma.

To our knowledge, this is the largest study to present TB-stigma community findings, unlike similar studies in the country that were patient-focused and facility-based or limited to a single district [3, 11, 14]. Consequently, the findings can support community-based TB program efforts such as the Community Awareness, Screening, Testing, and Prevention (CAST) and CAST-Plus campaigns. Moreover, the LQAS data can be further examined at administrative units to effectively bolster support for sub-divisions within districts [15, 18, 22, 23, 35]. Despite these strengths, the present study has limitations, although more than 50% of the districts in the country were covered, a complete country-coverage could provide additional insights. Moreover, the study relied on self-reported data, which could be influenced by social desirability bias [36]. However, the questions about stigma were not asked directly from the respondents’ perspective. Instead, they were framed in terms of the community’s beliefs or situation. This approach made it more likely to obtain honest responses. However, the cross-sectional design limitations of the initial (LQAS) survey [37] and the draw backs of secondary analyses [38] could not overcome by this study. For instance, while the questions used to assess stigma in the initial survey were similar to those in the standardized TB Stigma Assessment Tool developed by the STOP TB Partnership, there were some variations and they were not comprehensive [39]. Future LQAS survey should consider updating the TB module to reflect the standardized TB Stigma Assessment Tool developed by the STOP TB Partnership. Therefore, the results should be interpreted with these considerations in mind, along with any further necessary considerations.

## Conclusions

We found high levels of stigma with significant geographical and demographic disparities. Higher levels of stigma were reported in regions with varying levels of TB knowledge. TB stigma is more prevalent among older participants and those with secondary or higher education, aligning with previous research in Uganda. There was paradoxical relationship between TB knowledge and stigma, suggesting that TB knowledge interventions must balance increasing awareness with reducing fear. Community perceptions and deep-seated stigma were prevalent, these are shown to deter timely diagnosis and treatment, exacerbating the spread of TB. Therefore, targeted interventions are needed to change community attitudes and reduce discrimination against TB patients. Knowledge gaps and misconceptions about TB requires comprehensive educational programs to reduce both stigma and misinformation.

## Supporting information

S1 Checklist *PLOS ONE* clinical studies checklist. (DOCX)

S2 Checklist STROBE statement—checklist of items that should be included in reports of observational studies. (DOCX)

S1 Dataset (XLSX)

S1 File (XLSX)

S1 Fig Heat map showing TB stigma in Uganda. Spatial analysis of level of TB-related stigma and TB-related knowledge. Printed using QGIS under a CC BY license, with permission from Derrick Kimuli, original copyright 2024.
(TIF)
