# Supplementary figures and images for: Level of tuberculosis-related stigma and associated factors in Ugandan communities
Source: PLoS One. 2025 Jan 24;20(1):e0313750. doi: 10.1371/journal.pone.0313750 (PMC11761111; doi:10.1371/journal.pone.0313750)

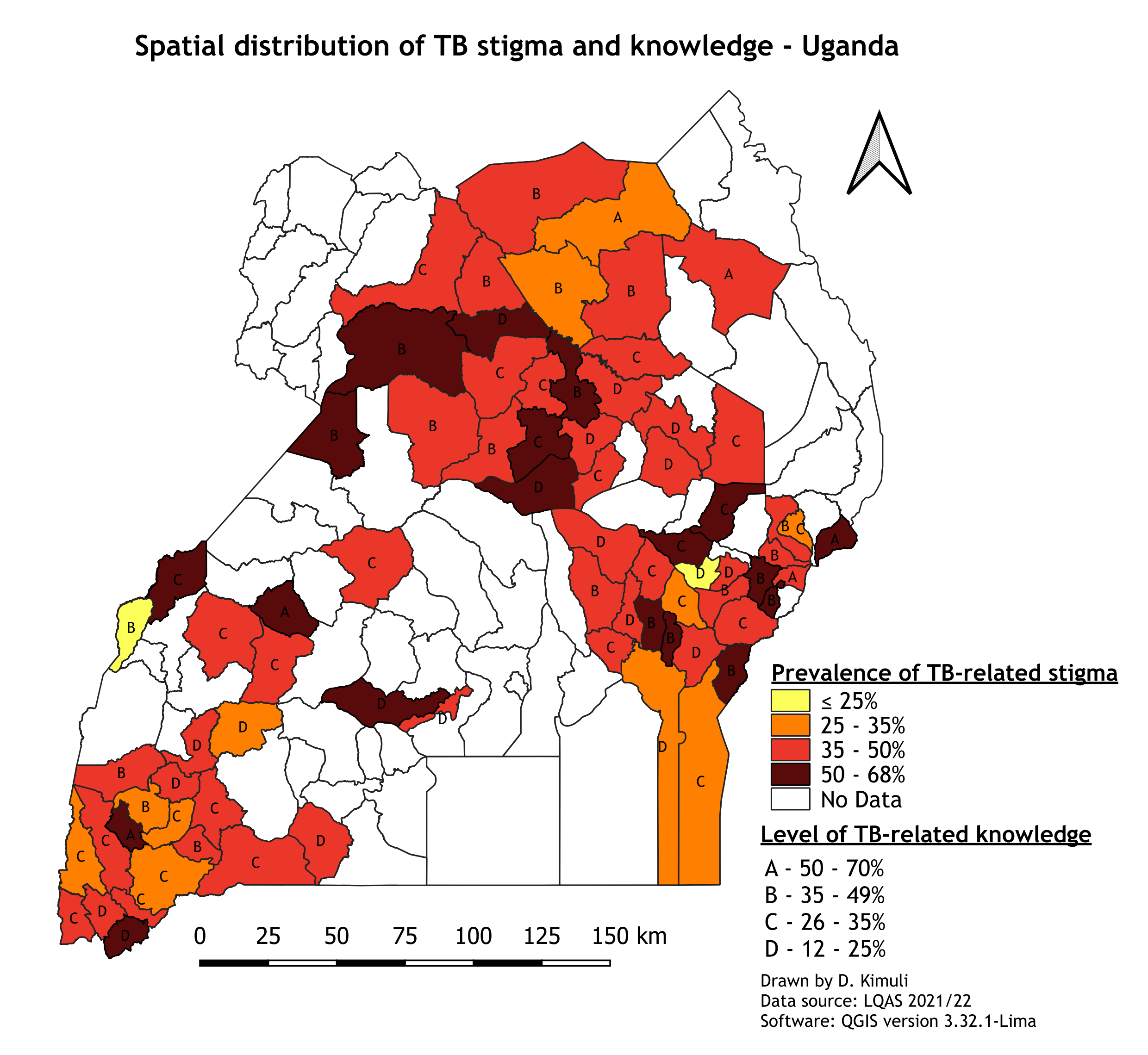

Supplement: S1 Fig — Spatial analysis of level of TB-related stigma and TB-related knowledge. Printed using QGIS under a CC BY license, with permission from Derrick Kimuli, original copyright 2024. (TIF) [file pone.0313750.s005.tif]
